# Approach-related anatomical differences in patients with lumbo-sacral transitional vertebrae undergoing lumbar fusion surgery at level L4/5

**DOI:** 10.1007/s00402-021-04303-2

**Published:** 2022-01-08

**Authors:** Luis Becker, Dominik Adl Amini, Katharina Ziegeler, Maximilian Muellner, Torsten Diekhoff, Alexander P. Hughes, Matthias Pumberger

**Affiliations:** 1 Center for Musculoskeletal Surgery, Charité-University Medicine Berlin, Charitéplatz 1, 10117 Berlin, Germany; 2 Department of Radiology, Charité-University Medicine Berlin, Charitéplatz 1, 10117 Berlin, Germany; 3 Spine Care Institute, Hospital for Special Surgery, 535 East 70th S, New York, NY 10021 USA

**Keywords:** LSTV, Lumbarization, Sacralization, ALIF, OLIF, LLIF

## Abstract

**Introduction:**

Lumbo-sacral transitional vertebrae (LSTV) are accompanied by changes in soft tissue anatomy. The aim of our retrospective study was to evaluate the effects of LSTV as well as the number of free lumbar vertebrae on surgical approaches of ALIF, OLIF and LLIF at level L4/5.

**Material and methods:**

We assessed the CTs of 819 patients. Of these, 53 had LSTV from which 11 had six (6LV) and 9 four free lumbar vertebrae (4LV). We matched them for sex and age to a control group.

**Results:**

Patients with LSTV had a higher iliac crest and vena cava bifurcation, a greater distance between the common iliac veins and an anterior translation of the psoas muscle at level L4/5. In contrast, patients with 6LV had a lower iliac crest and aortic bifurcation, no differences in vena cava bifurcation and distance between the iliac veins compared to the control group.

**Conclusions:**

For patients with LSTV and five or four free lumbar vertebrae, the LLIF approach at L4/5 may be hindered due to a high riding iliac crest as well as anterior shift of the psoas muscle. Whereas less mobilization and retraction of the iliac veins may reduce the risk of vascular injury at this segment by ALIF and OLIF. For patients with 6LV, a lower relative height of the iliac crest facilitates lateral approach during LLIF. For ALIF and OLIF, a stronger vessel retraction due to the deeper-seated vascular bifurcation is necessary during ALIF and is therefore potentially at higher risk for vascular injury.

## Introduction

Lumbosacral transition vertebrae (LSTV) are one of the most common congenital anomalies of the spine with a reported widespread prevalence of 4–36% in the population [1, 19]. They are defined by partial or complete lumbarization of the most cranial sacral vertebra as well as partial or complete sacralization of the most caudal lumbar vertebra. LSTV were first systematically classified by Castellvi et al*.* in 1984 [5].

A link between LSTV and increased lumbar back pain was assumed early on. On the one hand, constriction syndromes with nerve compression by the enlarged processus transversus, known as Bertolotti's syndrome, are considered [4, 11]. On the other hand, a higher rate of degenerative occurrences in the segment above is discussed in the literature [13, 15]. A lower mobility could be prevalent in LSTV segments with irregular articulation or partially bony fusion between the most caudal lumbar and the uppermost sacral vertebrae. This possibly results in a compensatory increased mobility of the segments lying above an LSTV and should may be taken into account as a cause of increased incidence of osteochondrosis, spondylarthrosis and disc degeneration [13, 17].

In cases where conservative therapy leads to unsatisfactory treatment results, therapy by fusion surgery for stabilization and indirect decompression of the neuroforamina should be considered [16]. Procedures coming from ventral retroperitoneal such as anterior lumbar interbody fusion (ALIF), lateral retroperitoneal approaches such as oblique (OLIF) or lateral lumbar interbody fusion (LLIF), dorsal procedures such as posterior (PLIF) or transforaminal lumbar interbody fusion (TLIF) as well as combined procedures with transpedicular screw fixation are commonly used for this purpose [16]. The literature shows that changes in bone anatomy in LSTV are accompanied by changes in soft tissue anatomy, which can significantly complicate approaches, especially for retroperitoneal procedures, in which the large abdominal vessels are exposed [3, 12, 17].

However, the literature has so far not taken into account the number and the degree of expression of transitional lumbar vertebrae when reviewing the feasibility and risks of lumbar spinal fusion procedures.

## Materials and methods

**Fig. 1:**
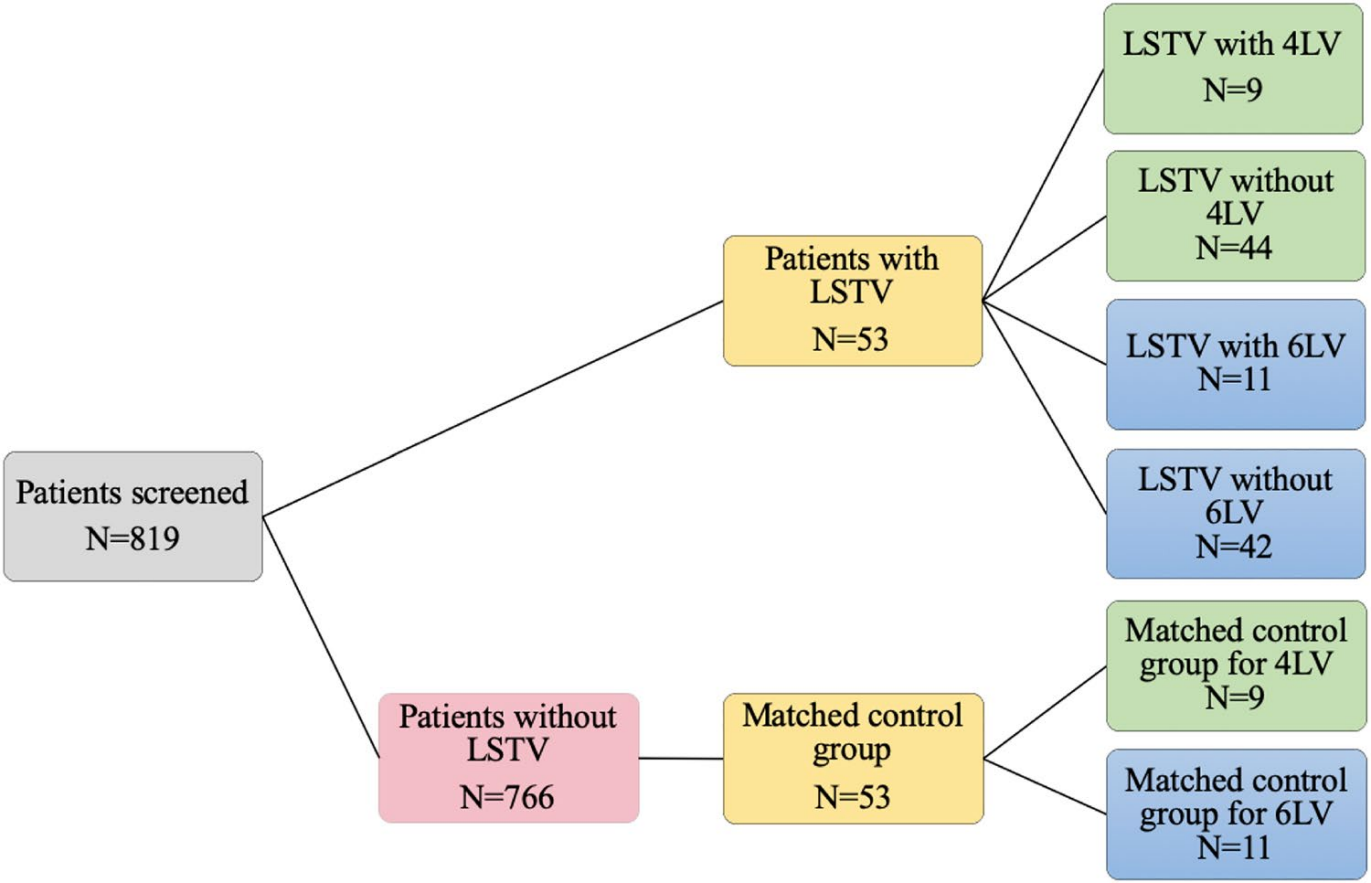
Flow chart diagram of patients cohorts

We assessed the feasibility of ALIF and OLIF or LLIF for patients with lumbo-sacral transitional vertebrae in patients with LSTV with regular number of free lumbar vertebrae, with bony lumbarization (6LV) or sacralization (4LV). Therefore, we performed a retrospective matched-pair analysis of abdomen-pelvic CT scans with groups as shown in Fig. 1. We assessed the retroperitoneal vessels, the relative location of the iliac crest and the musculus psoas in reference to the center of the ventral edge of the disc between the fourth and the fifth vertebra caudally to the rib bearing vertebrae (L4/5). The first vertebra without a rib below the rib-bearing vertebrae was counted as L1, the vertebrae were counted caudally. We counted sixth free lumbar vertebrae when a disc was found to be fully continuous between vertebral bodies of L6 and S1. Four free lumbar vertebrae were present when an osseous fusion of the fifth lumbar vertebra with the os sacrum was present.

LSTV were classified according to the Castellvi classification [5]. Patients classified as type I (a = unilateral, b = bilateral) show a dysplastically enlarged processus transversus > 19 mm without pseudarthrosis or osseous fusion with the vertebra below. In cases of pseudarthrosis of the processus transversus with the os sacrum, these are assigned to type II (a = unilateral, b = bilateral). Patients with unilateral fusion of the processus transversus with the os sacrum are assigned to type IIIa and patients with bilateral fusion to type IIIb. If there is a unilateral fusion and a contralateral pseudarthrosis between the transverse process and the os sacrum, they are classified as type IV.

### Individuals

The study was approved by the local ethics board (ethics proposal number EA1/300/19). We included patients retrospectively who underwent CT imaging of the abdomen and pelvis due to tumor staging, bleeding source detection, infectious focus search and trauma examinations in our Department of Radiology from 2016 to 2019. Slice thickness were 0.5 mm, images were reconstructed in a soft tissue kernel with beam-hardening compensation. We excluded patients with primary and metastatic malignancy of the musculoskeletal system, prior apparent surgery or fracture of the spine or pelvis, rheumatic diseases with apparent involvement of the sacro-iliac joint, and insufficient cross-sectional imaging by low-dose CT or missing image sections.

A total of 819 patients fit our inclusion criteria of which 53 patients had LSTV. We matched these 53 patients by propensity score matching with a tolerance of 0.01 for age and sex to a control group without LSTV from the remaining 766 patients. We compared the group with six free lumbar vertebral bodies (6LV) (*n* = 11) against a matched control group without LSTV (*n* = 11) and against all patients with LSTV but without six free lumbar vertebral bodies (*n* = 42). Similarly, we compared the group with four free lumbar vertebral bodies (4LV) (*n* = 9) against a matched control group without LSTV (*n* = 9) and against the patients with LSTV but without four free lumbar vertebral bodies (*n* = 44).

### Image assessment and measurements

**Fig. 2:**
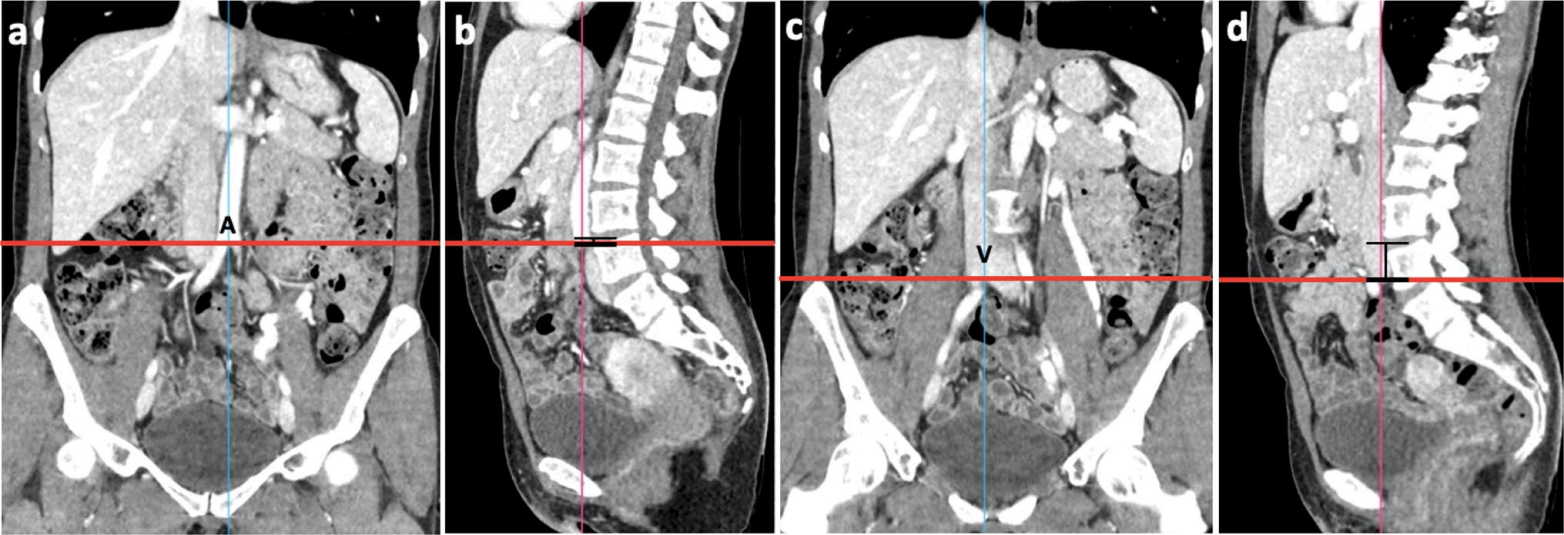
Measurement of the height difference between the aortic bifurcation (**a**,  **b**) vena cava bifurcation (**c**, **d**) and the disc space L4/L5. Vena cava is marked by V, aorta is marked by A

Assessment of whether LSTV were present and classification according to Castellvi were performed independently by two radiologists in consensus reading [5]. A resident orthopedic surgeon supervised by a senior consultant spine surgeon performed measurements. The image analysis software RadiAnt DICOM Viewer 2020.2 (Medixant, Poznan, Poland) were used for reconstruction and measurement of the following parameters. The extent and location of the psoas muscle in relation to the disc space at level L4/5 were evaluated and the parts in which it was partially present were counted and classified (anterior, posterior, anterior first quarter, second quarter, third quarter, fourth quarter). The level of the bifurcation of the aorta as well as the inferior vena cava was referenced to the midpoint of the anterior edge of the disc space L4/5 from the lowest point of the bifurcation, as shown in Fig. 2.

**Fig. 3:**
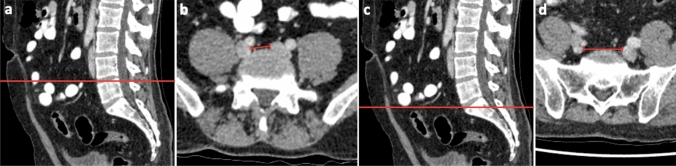
Measurement of the minimal distance between the iliac veins at level L4/5 (**a**,  **b**) and L5/S1 (**c**, **d**)

In addition, the minimum distance between the two venae iliacae communes or internae was measured at the reference level L4/5 and L5/S1, depending on whether the venae iliacae communes had already divided at the corresponding level as shown in Fig. 3. For this purpose, the shortest distance between the medial vein walls was measured in axial plane at level L4/5 and L5/S1.

**Fig. 4:**
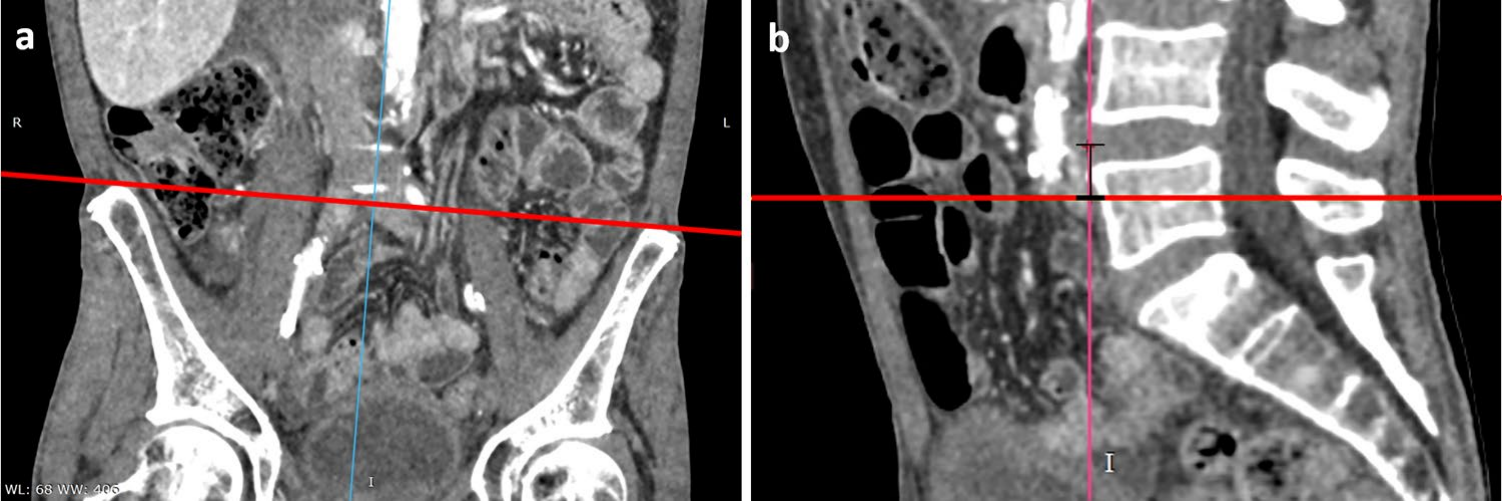
The Measurement of the distance of iliac crest in relation to disc space L4/5

The height of the iliac crest was assessed as shown in Fig. 4. As presented in Fig. 4a, the red line, which tangentially touches both iliac crests, was defined in the coronal plane. Figure 4b shows the measurement of the minimum distance between the tangential line defined in (a) and the reference point of the disc space L4/5. To minimize rotational errors due to patients lying oblique on the CT table for assessment of the height of the iliac crest, an axis that touched both iliac crests cranially tangentially was formed in the coronal plane passing through the ventral edge of the disc space L4/5. The shortest distance of this line to the center of the ventral edge of the disc space L4/5 was evaluated as the distance between the iliac crests and the disc space L4/5.

### Statistical analysis

For statistical analysis we used SPSS Version 27 (IBM Corporation, New York, NY, United States). To compare results between matched control groups Wilcoxon signed-rank test was used for numerical data. For ordinal scaled data Pearson's chi-squared test were used. A significance level of *p* < 0.05 was assumed for all tests.

## Results

### Patients

53 of the 819 patients evaluated had LSTV (6.5%; 53/819) (see Table 1), with eleven patients (1.3%; 11/819) having six free lumbar vertebrae and nine patients having four free lumbar vertebrae (1.1%; 9/819). There were no significant differences in age (mean 51.6 years, (range 16–81/86) (LSTV/control) *p* = 0.954) or sex (25/53 female in LSTV and control, *p* = 1.000) between the cohort of patients with LSTV and the matched control group.

**Table 1:** Grouping of patients with LSTV according to the Castellvi classification

	Castellvi type			
	I	II	III	IV
LSTV (*n*)	16	23	7	7

### Level of the iliac crest

The level of the iliac crest in relation to the midpoint of the ventral disc space L4/5 differed significantly between patients with LSTV and the control group as shown in Table 2. The number of free lumbar vertebrae also had a significant influence on the height of the iliac crest (see Table 2).

**Table 2:** Minimal distance between a line, intersecting tangential both cristae iliacae and disc space L4/5.

	Median (IQR)	*p*-value
LSTV	0.38 cm (± 2.29 cm)	**0.002**
Control	− 1.00 cm (± 1.52 cm)	
6LV	− 3.10 (± 1.87 cm)	**0.013**
Control	− 0.76 (± 0.88 cm)	
4LV	1.24 cm (± 1.61 cm)	**0.008**
Control	− 0.76 cm (± 0.90 cm)	

Significant differences are marked in bold

*IQR*  interquartile range

In addition, patients with 6LV differed not only in a significantly lower iliac crest compared to the control collective but also compared to the other patients with LSTV (*p* = 0.028; 6LV: − 3.10 cm (± 1.87 cm) median (IQR), LSTV without 6LV: 0.51 cm (± 1.68 cm)). Patients with 4LV did not show significantly changed level of crista iliaca in relation to disc space L4/5 compared to patients with LSTV with more than 4 free lumbar vertebrae (*p* = 0.500). Furthermore, gender had an influence on the height of the iliac crest. Women had a significantly lower iliac crest compared to men (*p* = 0.041, female: − 0.72 cm (± 2.24), male − 0.08 (± 2.06)).

### Level of aortic bifurcation

The presence of LSTV did not significantly affected the level of aortic bifurcation related to disc space of L4/5 compared to the control group. In both cohorts, the level of aortic bifurcation was significantly above the ventral midpoint of the L4/5 disc space as shown in Table 3. However, patients with 6LV had an aortic bifurcation that was on median 0.17 cm below the midpoint of the disc space L4/5 in contrast to the matched control group in which the aortic bifurcation was on median 2.08 cm above the midpoint of the disc space. In addition, patients with 6LV also differed compared to the remaining patients with LSTV but without 6LV (*p* = 0.046; 6LV: − 0.17 cm (± 1.38 cm), LSTV without 6LV 2.95 cm (± 1.68 cm)). Patients with 4LV did not show significantly changed height of aortic bifurcation in relation to disc space L4/5 compared to patients with LSTV with more than four free lumbar vertebrae (*p* = 0.686). No sex differences were detected for vascular anatomy (*p* > 0.05).

**Table 3:** Minimal distance between the aortic bifurcation and disc space L4/5

	Median (IQR)	*p*-value
LSTV	2.60 cm (± 2.51 cm)	0.054
Control	1.65 cm (± 1.67 cm)	
6LV	− 0.17 (± 1.38 cm)	**0.033**
Control	2.08 (± 2.94 cm)	
4LV	2.25 cm (± 2.44 cm)	0.260
Control	2.08 cm (± 2.81 cm)	

Significant differences are marked in bold

*IQR*  interquartile range

### Level of vena cava inferior bifurcation

Patients with LSTV in our cohort showed significantly higher inferior vena cava bifurcation with respect to the reference level L4/5 than the control group as shown in Table 4.

**Table 4:** Minimal distance between the vena cava inferior bifurcation and disc space L4/5

	Median (IQR)	*p*-value
LSTV	0.28 cm (± 3.02 cm)	**0.001**
Control	− 1.13 cm (± 1.91 cm)	
6LV	− 2.66 (± 3.14 cm)	0.091
Control	0.17 (± 1.90 cm)	
4LV	1.31 cm (± 1.97 cm)	0.086
Control	− 0.44 cm (± 2.87 cm)	

Significant differences are marked in bold

*IQR*  interquartile range

Only patients with 6LV differed significantly compared to patients with LSTV but without six free lumbar vertebrae by inferior vena cava bifurcation below the reference level L4/5 (*p* = 0.028; 6LV: − 2.66 (± 3.14 cm), LSTV without 6LV 0.83 cm (± 1.84 cm)).

### Distance of iliac veins on level L4/5, L5/S1

Concomitant with the higher bifurcation level of the patients with LSTV compared to the matched control group, they also had a significantly longer distance between the two venae iliacae communes at level L4/L5 (*p* = 0.001). At level L5/S1, no significant difference existed in the distance between the venae iliacae communes/internae between patients with LSTV and the control group. Patients with four as well as with six free lumbar vertebrae did not differ significantly from the control group in the distance between the venae iliacae, both at level L4/5 and at level L5/S1.

### Extent of the psoas muscle at level L4/5

The psoas muscle was stretched ventrally in patients with LSTV, so that it was located partially ventral to the L4/5 disc. In patients with LSTV, in 61.32% the psoas muscle was stretched anterior to disc space L4/5, whereas in the control group, the psoas muscle anterior to the disc space was present in only 45.28% of patients. In the anterior first (LSTV 83.02%, control 82.08) and second quarter of disc space L4/5 (LSTV 97.17%, control 100%), the two groups did not differ substantially in the frequency of the presence of psoas muscle. However, in the third quarter (LSTV 73.58%, control 95.28%) as well as in the posterior quarter (LSTV 50.00%, control 74.53%), the two groups differed substantially. Posterior to the disc segment L4/5, psoas muscle was present in almost similar frequency in both groups (LSTV 23.58%, control 22.64%).

## Discussion

Consistent with the literature, our study indicates that the anatomical changes associated with LSTV can have a significant impact on spine surgery [12, 17]. LSTV have bony alterations, whereby a partially asymmetric bony configuration of the LSTV could hinder pedicular screw fixation due to pedicle asymmetry [18]. Not only the spine but also the bony pelvis shows significant changes in patients with LSTV. Here, on the one hand, a significant influence on the fixed pelvic parameters with the pelvic incidence, on which the spinal alignment is based, has been reported [8]. On the other hand, the literature shows that procedures utilizing a lateral retroperitoneal transpsoas approach such as LLIF may be hampered by a more elevated iliac crest [12, 16]. In accordance with the literature, our study showed a sex difference with regard to pelvic morphology [21]. Women had a significantly lower iliac crest compared to men which may facilitate approach for LLIF at level L4/5. Consistent with the results of Josiah et al. our patients population with LSTV showed a significantly higher standing iliac crest in relation to the disc space L4/5 [12]. In contrast, the group with 6LV had a significantly more caudally seated iliac crest in relation to the L4/5 disc space compared to the control group. As a result, the feasibility of LLIF at level L4/5 could be impaired by the need for increased positioning and angulated exposure in patients with LSTV and a regular or decreased number of free lumbar vertebrae. However, approach in our collective of patients with 6LV was not hampered because of a lower seated iliac crest compared to the control group. In line with the literature, our study shows that a change in bony anatomy is accompanied by a change in soft tissue anatomy [3, 12, 17]. For an LLIF, the psoas muscle must be dissected for exposure of the intervertebral disc space. The recommended window to pass through the psoas muscle is at level L4/5 in the two ventral quarters in Moro Zones 1 and 2 due to the lumbar plexus [9, 22]. The literature reports possible changes in the lumbar plexus associated with LSTV [10]. This, accompanied by an anterior shifted psoas muscle could result in a higher risk for lumbar plexus injury in patients with LSTV performing an LLIF approach [2, 14, 20].

Our study confirmed the findings of Josiah et al. and Moreau et al. that LSTV have an impact on the anatomy of the prevertebral large abdominal vessels [12, 17]. In OLIF as well as ALIF, the left common/internal iliac artery and vein or the venous and aortic bifurcation must be mobilized and retracted for the exposure of the disk space and placing of implants [7, 16, 22]. This leads to a risk for vascular injury, especially from exposure of the L4/5 disc space [7, 22]. In line with the results of Josiah et al. reporting a 1.09 cm more cranialized bifurcation of the inferior vena cava relative to the midpoint of the ventral edge of the L4/5 disc level in patients with LSTV compared with a control group, and Moreau et al. reporting a 1.08 cm more cranialized bifurcation, in our cohort the bifurcation of the inferior vena cava was elevated by a median of 0.95 cm in patients with LSTV [12, 17]. Concomitantly, our patient population with LSTV had a significantly larger portal between the common iliac veins at the L4/5 level, requiring less mobilization and retraction of the veins for cage insertion in ALIF or OLIF, thereby a risk reduction for vascular injury should be considered [6]. Consistent with the findings of Josiah et al., our collective with LSTV tended to have cranialized aortic bifurcation compared with the control group, though this did not reach the level of significance [12]. Patients with 6LV differed significantly from the other LSTV patients and had a significantly caudalized aortic bifurcation compared with the control population as well as a vein bifurcation that also tended to be more caudal, though the significance level was not reached. This may increase the risk of vascular injury for ALIF or OLIF in patients with 6LV by an increased need for vessel mobilization and retraction.

Some limitations need to be mentioned. Due to the CT sectional imaging on which the study is based, the lumbar plexus in the psoas muscle can be visualized only to a limited extent and thus no definitive statement can be derived about the risk of injury to the nervous structures. The CTs were performed on the basis of different indications. In some cases, a contrast medium was applied, which contrasted the vessels and thus a difference in the appearance of the vessels existed. Numerical changes in the number of lumbar vertebrae, according to the literature, also did not have a large prevalence in our collective, so the group size of 4LV and 6LV was limited.

## Conclusions

In patients with LSTV, ALIF, LLIF or OLIF can also be safely performed at level L4/5. To reduce the risk of serious intraoperative complications such as severe bleeding or nerve injury, LSTV should be accurately diagnosed, anatomical sex differences should be taken into account and the appropriate procedure selected for each patient. Patients with LSTV and four (4LV) or five free lumbar vertebrae had a significantly high riding iliac crest and a reduction of the cross-sectional area of the psoas muscle in our collective, potentially hindering the feasibility of LLIF at level L4/5. However, they also showed a cranialized bifurcation of the vena cava and a wider venous portal, so ALIF or OLIF may be associated with a reduced risk of vascular injury. Patients with six free lumbar vertebrae had a deeper-seated iliac crest therefore the approach for LLIF is not hampered. Because of a caudalized vascular bifurcation, there could be an increased risk of vascular injury due to the increased retraction required when performing an ALIF or OLIF.
